# Possible Hepatoprotective Effect of Tocotrienol-Rich Fraction Vitamin E in Non-alcoholic Fatty Liver Disease in Obese Children and Adolescents

**DOI:** 10.3389/fped.2021.667247

**Published:** 2021-07-08

**Authors:** Farah D.R. Al-Baiaty, Aziana Ismail, Zarina Abdul Latiff, Khairul Najmi Muhammad Nawawi, Raja Affendi Raja Ali, Norfilza Mohd Mokhtar

**Affiliations:** ^1^Department of Physiology, Faculty of Medicine, Universiti Kebangsaan Malaysia, Kuala Lumpur, Malaysia; ^2^Department of Pediatrics, Faculty of Medicine, Universiti Kebangsaan Malaysia, Kuala Lumpur, Malaysia; ^3^Gastroenterology Unit, Department of Medicine, Faculty of Medicine, Universiti Kebangsaan Malaysia, Kuala Lumpur, Malaysia; ^4^GUT Research Group, Faculty of Medicine, Universiti Kebangsaan Malaysia, Kuala Lumpur, Malaysia

**Keywords:** non-alcoholic fatty liver disease, vitamin E, tocotrienol, obesity, children, overweight

## Abstract

Obesity has become a worldwide health concern among the pediatric population. The prevalence of non-alcoholic fatty liver disease (NAFLD) is growing rapidly, alongside the high prevalence of obesity. NAFLD refers to a multifactorial disorder that includes simple steatosis to non-alcoholic steatohepatitis (NASH) with or devoid of fibrosis. NAFLD is regarded as a systemic disorder that influences glucose, lipid, and energy metabolism with hepatic manifestations. A sedentary lifestyle and poor choice of food remain the major contributors to the disease. Prompt and timely diagnosis of NAFLD among overweight children is crucial to prevent the progression of the condition. Yet, there has been no approved pharmacological treatment for NAFLD in adults or children. As indicated by clinical evidence, lifestyle modification plays a vital role as a primary form of therapy for managing and treating NAFLD. Emphasis is on the significance of caloric restriction, particularly macronutrients (fats, carbohydrates, and proteins) in altering the disease consequences. A growing number of studies are now focusing on establishing a link between vitamins and NAFLD. Different types of vitamin supplements have been shown to be effective in treating NAFLD. In this review, we elaborate on the potential role of vitamin E with a high content of tocotrienol as a therapeutic alternative in treating NAFLD in obese children.

## Introduction

The primary reason for chronic liver disease among children can be attributed to non-alcoholic fatty liver disease (1). NAFLD emerges as a common condition, usually accompanied by obesity, with males having a higher risk of developing the disease (2). NAFLD is described as hepatic fat infiltration of more than 5% of liver weight with no underlying liver diseases. It constitutes a series of conditions, ranging from intrahepatic fat accumulation to necrotic fibrosis and inflammation (3). The disease is generally asymptomatic, yet if left untreated, it will eventually lead to liver cirrhosis and, in some cases, hepatocellular carcinoma (4). In children, NAFLD is linked with metabolic impairments, which considerably increases the risk of metabolic and cardiovascular complications. Obesity is a central player in the development of metabolic syndrome, which is also highly related to NAFLD progression. Research has noted that many non-communicable ailments such as type II diabetes mellitus (T2DM), cardiovascular diseases, and cancers are also associated with obesity, which could further endorse the burden of illnesses and death rates (5). Obesity occurring in childhood has short-term and long-term effects on physical health in a child as well as in adult life. According to the previous research, obese and overweight children were more likely to stay obese in adulthood (6). Obesity occurring in childhood is an intricate issue, and it is the outcome of interactions between many aspects. These aspects can be split into two classes: changeable aspects (e.g., physical activity, socioeconomic status, diet, and parental elements) and unchangeable factors, including genetics, ethnicity, intrauterine components (7). For instance, Partap et al. suggested that obese and overweight children in Malaysia were highly associated with parental obesity where children of one or both obese parents had a 2-fold risk of being obese (8).

According to the National Health and Morbidity Survey (NHMS) 2015, the prevalence of obesity among children aged 10–14 years in Malaysia was 14.4% (9). While in the “MyBreakfast” study, the prevalence of overweight and obesity among children aged 6–12 years was 14.7% in Malaysia (10). Likewise, the NHMS conducted in three separate years 2006, 2011, and 2015, documented an uprising in the prevalence of obesity and overweight among Malaysian youths of <18 years old at 5.4, 5.7, and 11.9%, respectively (9, 11, 12). The rise in the rate of overweight or obese children could increase NAFLD prevalence among pediatrics. Risk factors, including, including insulin resistance, high body mass index (BMI), and genetic predisposition of NAFLD, should alert the parents or guardians to screen for NAFLD among children (13). Based on the NAFLD guidelines by the North American Society Pediatric Gastroenterology, Hepatology and Nutrition (NASPGHAN), overweight or obese child above 8 years old with chronically elevated alanine aminotransferase (ALT) and identified risk factors should be screened for NAFLD. The NASPGHAN suggested intervening against the disease at a younger age, especially those with a greater risk of certain subpopulations, including ethnicity and presence of metabolic syndromes (14). To date, there is no pharmacological treatment for NAFLD, both in adults and children. The first-line therapeutic approach for NAFLD among children is lifestyle changes, including diet and exercise (15). A weight loss of 5–10% of the original body weight is recommended to treat NAFLD (16). Interestingly, Cheng et al. showed a pronounced reduction in hepatic fat content in pre-diabetic adult patients with NAFLD who received a combination of fiber-enriched diet and aerobic exercise training compared to patients receiving either increased fiber-intake or exercise alone (17). A Mediterranean diet is a promising approach to the treatment of NAFLD. A significant decrease in fat deposition in the liver was observed among overweight adult patients with NAFLD after 6 months of intervention (18). Likewise, Corte et al. showed a lowered risk of non-alcoholic steatohepatitis (NASH) and diabetes in obese pediatric patients who adhered to the Mediterranean diet compared to children and adolescents who did not (19). In a randomized controlled trial of 40 adolescent boys, Schwimmer et al. reported a significant decrease in hepatic steatosis and ALT level in the intervention group receiving a diet of free sugar intake to <3% of daily calories. The finding suggested the provision of a diet low in free sugar for a better disease outcome (20). Besides, epidemiological studies demonstrated that children and adolescents with NAFLD consumed a higher amount of fructose intake than healthy controls. Therefore, reduced consumption of high-sugar beverages could prevent NAFLD (21).

Until today, there is no FDA approved drug in managing NAFLD among children. The safer choice of treatment to tackle this disease was probiotics or antioxidant agents (22). The rationale of using an antioxidant agent such as vitamin E was based upon the fact that oxidative stress is one of the possible underlying mechanisms that have a role in the progression of liver steatosis to fibrosis. The imbalance of antioxidants and production of reactive oxygen species (ROS) occurred during fatty acid β-oxidation and oxidative phosphorylation which lead to hepatic damage and injury (23). Moreover, an excessive amount of ROS could trigger the production of pro-inflammatory cytokines hence exacerbating the disease (24). This review summarizes the insights on NAFLD occurring among children and the use of vitamin E, a well-established fat-soluble antioxidant, in the treatment for obese pediatric NAFLD (25).

## Pediatric Non-Alcoholic Fatty Liver Disease (NAFLD)

### Epidemiology

The prevalence of NAFLD was closely related to the increased prevalence of obesity in adults and children (26). The occurrence of obese and overweight children has attained epidemic levels in several emerging nations, including Malaysia. Overweight and obese children had a higher risk of NAFLD, with an estimated prevalence of 26.0% in children with obesity (27). In a recent meta-analysis, the global prevalence of NAFLD among adults was estimated to be at 25.24% (4). The prevalence of NAFLD in Asia among adults increased significantly from 25.28% in 1999–2005 to 28.46% in 2006–2011, and 33.9% in 2012–2017 with higher prevalence observed among obese to non-obese populations in most Asian countries (28). Anderson et al. performed a meta-analysis on NAFLD among youths and estimated the prevalence of NAFLD in children from the general population was at 7.6% (95% CI: 5.5–10.3%), while from 56 independent studies in child obesity clinics, the prevalence was much higher at 34.2% (95% CI: 27.8–41.2%) (2). It is projected for the next 10 years; the prevalence of pediatric NAFLD would emerge as the most widespread reason for liver failure and a warning for liver transplantation in kids and adolescents (2, 29).

The prevalence of NAFLD increases with age, with a greater percentage of NAFLD among adolescents (15–19 years old) at 17.3% as compared to toddlers (2–4 years old) at 0.7% (30). NAFLD increased occurrence in adolescents could be attributed to several changes occurring during childhood to adulthood, including variation in diet, hormone levels, and metabolism (31). The prevalence rate of NAFLD was 13.4% (278/2,080) in boys, which was significantly higher than girls (2.8%, 57/2,061) (32). A probable hypothesis was that gender disparity in NAFLD is potentially due to differences in sex hormones and that females are more resistant to NAFLD progression than males due to the presence of estrogen, which potentially has a liver-protective effect (33). However, the role of gender in influencing the prevalence of NAFLD remains a question. Other factors that contribute to pediatric NAFLD were the sedentary lifestyle of children and their unhealthy food choices (34). In the United States, the prevalence of NAFLD, was also affected by ethnicity based on a meta-analysis study of 34 studies, in which a higher risk of NASH was observed in Hispanics compared to Caucasians and African Americans (35). Likewise, among adolescents with NAFLD, a higher prevalence was recorded among Hispanics (59.6%) compared to Caucasians (42.9%) and African Americans (15.7%) (36). In Malaysia, of 469 adult subjects analyzed, a higher prevalence of NAFLD was observed among Indian (33.3%) and Malay (25.5%) males compared to Chinese males (6.8%) (37). The ethnic differences among NAFLD patients could probably be explained by an increased insulin resistance where Asian Indians were reported to have the least insulin sensitivity compared to Chinese or Malay males (38). In a cross-sectional study on the impact of insulin resistance and hypertriglyceridemia, non-Hispanic Whites and African Americans displayed greater insulin sensitivity compared to Hispanic White, East Asian, and South Asian (39). Pediatric NAFLD had a strong association with central or generalized obesity and metabolic syndromes, including insulin resistance and dyslipidaemia (14). In children and adolescents with biopsy-proven NAFLD, a higher prevalence of abnormal glucose tolerance was observed than children without NAFLD. Ting et al. reported a significant association between metabolic syndrome and advanced liver fibrosis in 57 children visiting pediatric obesity and diabetic clinics (40). The severe form of NAFLD, NASH, was detected more among children with abnormal glucose tolerance (41). Mohamed et al. reported that 62% of obese children with NAFLD had metabolic syndrome (42).

Genetic predisposition is another factor that could affect the prevalence and severity of both adult and pediatric NAFLD. In Korea, an additive effect of single nucleotide polymorphisms (SNPs) was noted in the patatin-like phospholipase domain containing three genes (*PNPLA3*) and transmembrane six superfamily 2 (*TM6SF2*) that affect the severity of liver damage as reported in adult patients with NAFLD (43). In a PANIC study, overweight Caucasian children (6–8 years) with *PNPLA3*, I148M polymorphisms were associated with an increased ALT, indicating early onset of NAFLD (44). The *PNPLA3* polymorphisms among obese young individuals (below 20 years) with metabolic syndrome were shown to have a significant increase in their ALT levels (45). A meta-analysis study of pediatric and adolescents suggested that carriers of the G allele of *PNPLA3* have a more severe type NASH together with a high level of liver fat content, ALT, aspartate aminotransferase (AST), and gamma-glutamyl transferase (GGT) (46). In an association study investigating the relationship between *PNPLA3* and hepatic fat accumulation among children, Stanislawski et al. found hepatic fat accumulation is highly associated with *PNPLA3* among Hispanic compared to non-Hispanic (47). On the other hand, Grandone et al. reported that obese children carrying *TM6FS2* E167K were more predisposed to NAFLD (48).

### Pathogenesis

**Figure 1:**
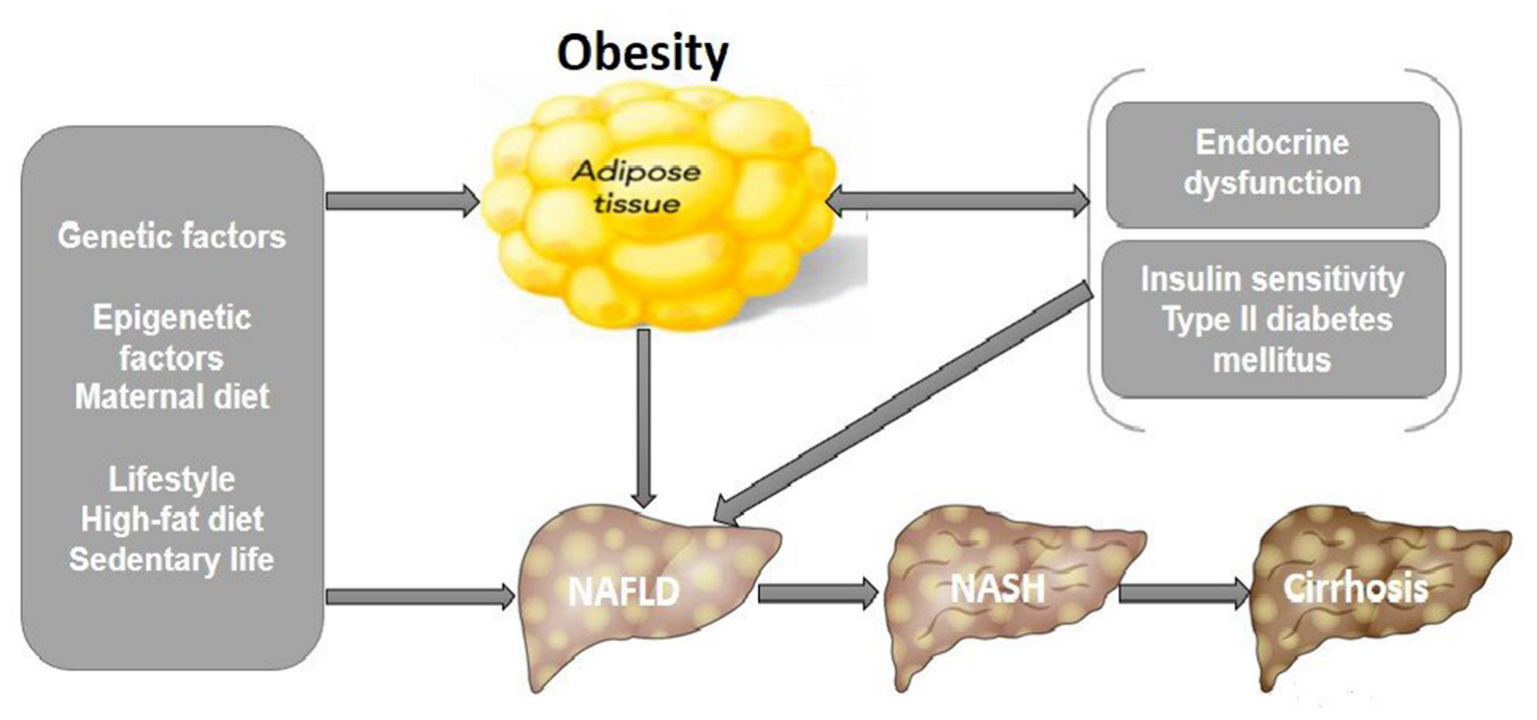
Pathophysiological mechanisms that link pediatric NAFLD and obesity. Unhealthy lifestyles, genetic and epigenetic factors are responsible for NAFLD and its progression to NASH and hepatic cirrhosis, increasing the risk of mortality. Proposed pathogenic mechanisms include endocrine dysfunction, hepatic insulin resistance from T2DM, and obesity increases lipid accumulation and inflammation in the liver leading to NAFLD. NAFLD, non-alcoholic fatty liver disease; NASH, non-alcoholic steatohepatitis; T2DM, type II diabetes mellitus (29).

The underlying mechanism in the pathogenesis of NAFLD is complex and involves many factors. Initially, the “two-hit theory” emerged where an accumulation of lipids due to a sedentary lifestyle, high fat diet, insulin resistance, and obesity act as the first hit. This was followed by the second hit theory of fatty acid accumulation that induced inflammatory cascades resulting in fibrosis (49). Nonetheless, a more recent “multiple-hit theory” better describes the intricacy of the disease where multiple parallel factors are involved in NAFLD progression. The “multiple-hits” hypothesis primarily elaborates on the NAFLD pathogenesis, which includes genetic factors, insulin resistance (IR), hyperlipidaemia, and obesity as the main culprits in the development of NASH (50). At the onset of NAFLD, it is characterized by insulin resistance and triglycerides (TGs) accumulation in the liver termed as steatosis. Factors, including hypercaloric diets, epigenetics, genetic susceptibility, and a sedentary lifestyle, significantly promoted NAFLD pathogenesis. Besides liver damage, this condition could lead to other related comorbid diseases. Nevertheless, the relationship and interactions between these mechanisms are not yet well understood (51). The fatty liver condition is non-pathogenic for most patients and is reversible through appropriate interventions, while 30% of them have the probability of progressing to NASH (52)^.^ The differences in storing fat capability in different adipose tissue compartments differed significantly between children with and without NAFLD (53). These dissimilarities were visible after 3 years of age but not at birth, indicating that the initial 3 years of life might signify a vital window, in which different interactions between environmental, genetic, epigenetic, and metabolic aspects lead to the prospective risk of primary NAFLD (54) (Figure 1) (29). Yet, Wesolowski et al. suggested the offspring of obese mothers may have increased risk for obesity and NAFLD at birth, in which the intrauterine aspects played a role in the rapid progression of NAFLD to NASH in children (55). In terms of metabolic aspect, insulin sensitivity is considered one of the hallmarks of NAFLD, is strongly associated with obesity. The impaired ability of insulin to suppress endogenous glucose and free fatty acids (FFAs) production is indicative hepatic insulin resistance (56). It was also suggested that IR was the result of an accumulation of excess fat that cannot be stored in the adipose tissue, which overflows into the visceral compartment and accumulates in organs such as the liver (57). On the other hand, secondary NAFLD may develop due a myriad of other causes including endocrine diseases such as primary hypothyroidism, hypogonadism, polycystic ovary syndrome (PCOS) and growth hormone deficiency which resulted in obesity (58).

Fatty liver is the build-up of TGs in hepatocytes due to a disparity between energy metabolism and energy consumption (59). Dyslipidaemia, which is a component of metabolic syndrome, is also one of the hallmarks of pediatric NAFLD. It is characterized by the accumulation of TGs and low high-density lipoprotein cholesterol (HDL-C). Reduced fatty acid β-oxidation in lipid degrading organelles (e.g., mitochondria and peroxisomes) and a decreased lipid excretion of very-low-density lipoproteins (VLDL) were other factors that contribute to the accumulation of hepatic fat (60). In turn, the adipose tissue production contributes to the increased in FFAs turnover. In a retrospective study of 309 children diagnosed with NAFLD, elevated levels of TGs, non-HDL-C and low-density lipoprotein cholesterol (LDL-C) were highly prevalent (60, 61). Local data documented that high TG was a predictor for NAFLD among overweight and obese children attending the Pediatric Obesity Clinic at the University Malaya Medical Center (42). In addition, increased TG synthesis could also be contributed by *de novo* lipogenesis in the liver. For instance, the conversion of fructose to fat in the liver further drives the accumulation of liver fat (62). Schwarz et al. reported that short-term isocaloric fructose restriction in children with obesity and metabolic syndrome resulted in significant decreased liver fat and *de novo* lipogenesis as well as improved insulin kinetics (63). Other factors contributing to NASH progression include lipotoxicity-induced inflammation and oxidative stress (59, 64). The ectopic fat accumulation may induce excessive release of pro-inflammatory cytokines such as TNF-α and IL-6, thus worsening the disease condition. Adipocytokines (or adipokines), secreted by the adipose tissues, were shown to be highly involved in various processes such as inflammation, immunity, insulin sensitivity, simple liver steatosis, and NASH (65). Studies have shown serum levels of adiponectin were altered in patients with NAFLD. A prospective case-control study suggested elevated serum chemerin and decreased serum adiponectin were highly associated with an increased NAFLD likelihood in non-diabetic obese children (66). In a prospective study of 3 years among adults without fatty liver, similar observation of decreased adiponectin level was observed among subjects who developed NAFLD (67). These novel adipokines (e.g., omentin and chemerin) have been suggested as potential markers of ectopic lipid accumulation in the liver and NAFLD pathogenesis (68). Pediatric NAFLD progression is illustrated through a complicated sequence of interactions between resident hepatic and recruited cells. The activation of Kupffer cells (liver macrophages) and hepatic stellate cells (collagen-producing cells) influenced NAFLD's progression to NASH. The activation of Kupffer cells led to the production of inflammatory cytokines as a direct outcome of apoptosis, hepatic steatosis, hepatocyte injury, and indirect reaction to hepatic damage (69, 70). Dysregulation of pro-inflammatory adipokines and cytokines were found to be globally prevalent in NAFLD patients. Mitochondrial, endoplasmic reticulum, cytokine-mediated oxidative stress, and hepatocytic apoptosis were the causes of NASH (71, 72).

**Figure 2:**
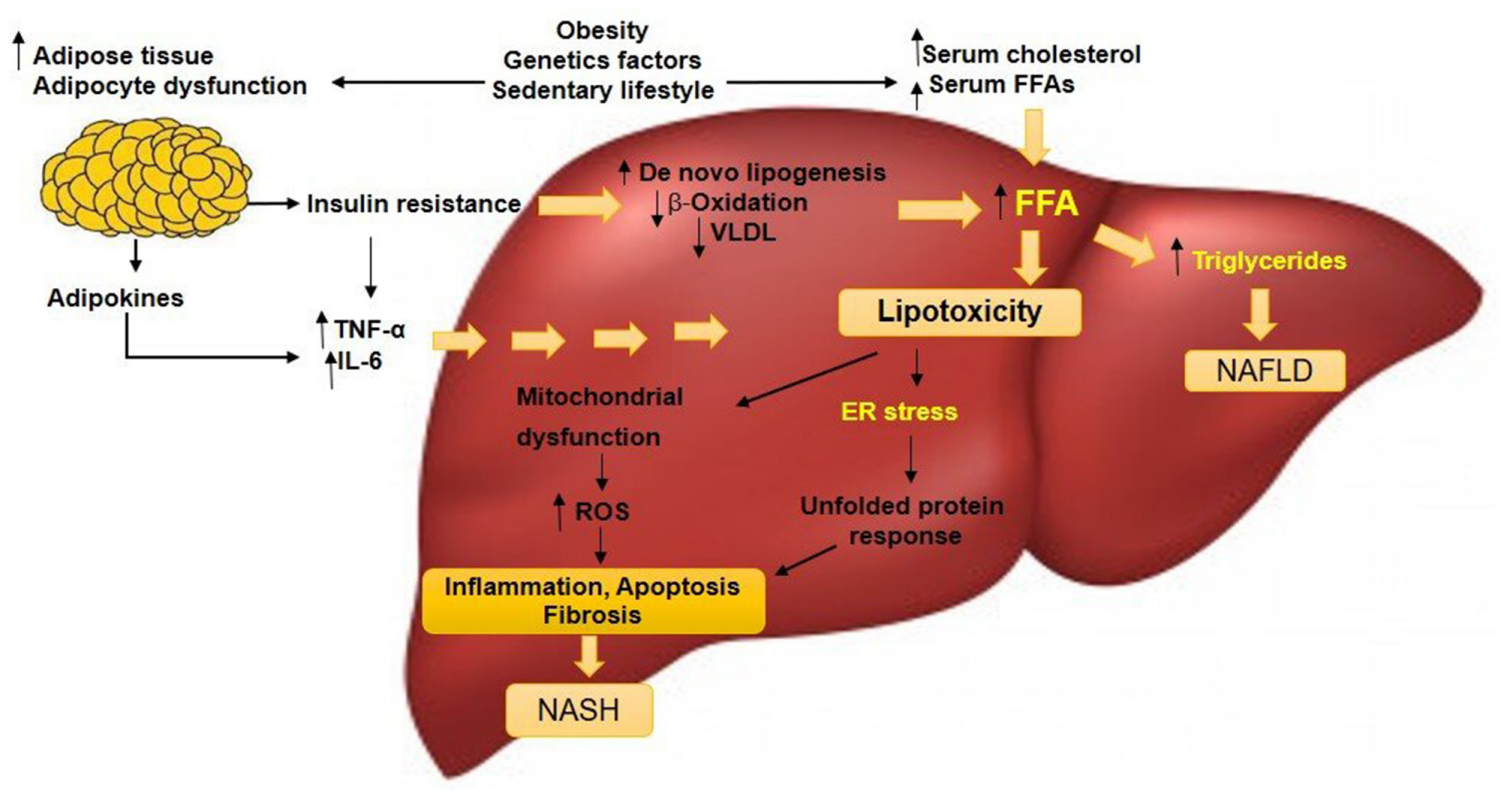
A simplified version of the pathogenesis of pediatric NAFLD. Factors including obesity, sedentary lifestyle, and genetic factors could increase serum levels of FFAs and cholesterol, stimulate adipocyte proliferation and dysfunction. Insulin resistance will develop and this condition acts on adipose tissue that deteriorates the adipocyte dysfunction. Lipolysis is induced, adipokines and pro-inflammatory cytokines such as TNF-α and IL-6 in the liver are released. Insulin resistance amplifies *de novo* lipogenesis and reduces β-oxidation and VLDL level, generating excessive FFAs. The increased hepatic FFAs leads to two situations: (1) synthesis and accumulation of TGs leading to persistent NAFLD and (2) increased 'toxic' levels of FFAs and free cholesterol, causing mitochondrial dysfunction, evidenced by an elevated ROS and ER stress. Hepatic inflammation occurs that progress to NASH. NAFLD, non-alcoholic fatty liver disease; NASH, non-alcoholic steatohepatitis; FFAs, free fatty acids; VLDL, very-low-density lipoproteins; ROS, reactive oxygen species; ER, endoplasmic reticulum; TNF-α, tumor necrosis factor-alpha; IL-6, interleukin 6. (50).

Obesity can also be defined as low-grade inflammation in adipose tissue, which resulted in IR and other metabolic complications associated with obesity (73). Inflamed adipose tissue was associated with abnormal productions of inflammatory cytokines and activation of inflammatory signaling in adipocytes (74). Furthermore, extracellular and intracellular stress induced by the accumulation of lipids, glucose, and cytokines and steatosis could trigger the hepatic endoplasmic reticulum (ER) stress response. The progression of steatosis to NASH could be due to the hepatic ER stress response's activation, triggering the cell death and inflammation, hence promoting metabolic disorders (75). However, there are limited studies on the association of inflammatory markers with NAFLD. Figure 2 illustrates the mechanisms involving multiple factors implicated in the pathogenesis of NAFLD to NASH. (50).

### Diagnosis

Pediatric NAFLD continues to be an underdiagnosed condition due to the dearth of symptoms' recognition and under-appreciation of its related complications. There is no specific screening strategy for NAFLD in children, even though there are clinical and pathophysiological variations between adult and pediatric NAFLD. Most diagnostic algorithms and risk prediction scores, including NAFLD activity scores, are used for adults but are limited for children; therefore, they could not be relied on (76, 77). In place of a procedure is excluded, NAFLD diagnosis needs to be actively considered in all obese or overweight children more than 10 years old, specifically in the contextual of acanthosis nigricans, hypertension, and evidence of hepatomegaly as clinical evidence of metabolic syndromes (29, 77, 78). Liver biopsy is the current benchmark approach for NAFLD diagnosis, and it is the sole mode of distinguishing between NASH and simple steatosis. It also could exclude other causes of elevated transaminases and hepatic infiltration due to fats such as autoimmune hepatitis and metabolic liver diseases (79). Nevertheless, liver biopsy is an invasive process with several drawbacks, such as high cost and risks, and hence it is often not carried out as a preliminary step for diagnosis (79). Therefore, a non-invasive evaluation in imaging tests, biochemical parameters, and serum biomarkers is used to assert the diagnosis of fatty liver disease among children, particularly in a typical patient with metabolic syndromes (80). Liver function and abdominal ultrasonography tests are the choices for diagnosing NAFLD among youth despite their narrow sensitivities (29, 80). As suggested by the American Academy of Pediatrics, regular semi-annual screenings for serum AST and ALT must be conducted for every obese child above 10 years of age, a BMI score ranged from the 85th percentile to the 94th percentile, and who possesses related metabolic risk factors (14). The European Society of Pediatric Gastroenterology, Hepatology, and Nutrition (ESPGHAN) suggested that all overweight and obese children above 3 years old should be screened for NAFLD by performing abdominal ultrasound and liver function tests. Ezaizi et al. suggested using a combination of elevated ALT and fatty infiltration on ultrasound to increase the detection rate and to avoid missed detection of suspected NAFLD children at risk (81). Ultrasonography is a safe and reliable tool for the diagnosis of NAFLD. Nonetheless, the hepatic ultrasound is insensitive when the fatty liver is moderate to severe as frequent mild level of hepatic steatosis was reported in advanced pediatric NASH. Therefore, liver biopsy is usually necessary for accurate pediatric NAFLD diagnoses. Histopathological and radiological findings are essential and must be interpreted cautiously, while serum ALT and AST levels are standard procedures in most pediatric NAFLD cases, regardless of the severity of the disease (29).

## Beneficial Effects of Vitamin E (Tocotrienol) in Non-Alcoholic Fatty Liver Disease

Presently, no therapeutic strategy was approved for NAFLD, both in adults and pediatrics. Lifestyle modification via diet and exercise was shown to benefit patients with NAFLD (82). Consequently, most clinical efforts are focused on treating the elements of metabolic syndrome, specifically obesity, hypertension, diabetes, and dyslipidaemia. Other interventions focused on specific pathways potentially implicated in NAFLD pathogenesis, including insulin resistance, apoptosis, pro-inflammatory cytokines, oxidative stress, angiotensin pathways, and bacterial overgrowth (83). In metabolic syndrome, the excess amount of FFA intake in the liver induces oxidative stress hence creating ROS that can cause liver damage. Thus, an antioxidant is an appropriate approach for adjunct therapy to lifestyle modification in treating NAFLD. Furthermore, Stenzel et al. analyzed the serum antioxidant level in metabolically healthy and unhealthy obese adolescents. They found a strong negative association between vitamin E and the waist circumference of unhealthy obese adolescents. In addition, lower frequency of NAFLD was documented among metabolically healthy obese subjects (84). The National Institute for Health and Care Excellence (NICE) and the American Association for the Study of Liver Diseases (AASLD) in their guidelines proposed using vitamin E in NAFLD patients, but the AASLD only approved vitamin E as a pharmacological treatment among adults without diabetes and with biopsy-proven NASH (85). Clinical trials testing the efficacy of vitamin E in both adults and pediatric NAFLD reported contradictory findings. In a meta-analysis by Amanullah et al. it was reported that adjuvant vitamin E therapy improved biochemical and histological characteristics in adult patients with NAFLD (86). However, in pediatric patients, the efficacy of vitamin E was moderate when compared to the placebo. An earlier studies that looked at the effect of vitamin E in children with NAFLD found that antioxidant therapy of α-tocopherol 600 IU/day and ascorbic acid 500 mg/day did not have significant effect on the liver function and glucose control (87). The authors suggested that diet and physical exercise are necessary for improving liver function and glucose metabolism in children with NAFLD. Notably, in a randomized, double-blind placebo-controlled trial between 80 adolescents with biopsy-proven NAFLD receiving either placebo or vitamin E and hydroxytyrosol (HXT), the results showed improved insulin resistance, steatosis, and oxidative stress parameters in children with NAFLD in the intervention arm (88). The use of vitamin E in combination with other nutraceuticals as a treatment in pediatric NAFLD showed promising results. About 70 children with NAFLD receiving vitamin E (10 mg) and hydroxytyrosol (7.5 mg) for a duration of 4 months showed significant reduction in TG level and improved liver steatosis (89). Zöhrer et al. treated 60 children (4–16 years) with liver biopsy-proven NASH with a combination of 250 mg of docosahexaenoic acid (DHA), 39 UI of vitamin E, and 201 mg of choline (DHA-CHO-VE) for 12 months. Significant improvement of liver steatosis was observed in the treated group accompanied by reduced ALT and glucose levels (90)^.^ Administration of vitamin E (400 IU/day) plus spironolactone (25 mg once daily) for 52 weeks showed a significant decrease in liver fat score and homeostasis model of assessment-insulin resistance (HOMA-IR) in women with NAFLD (91).

Vitamin E is a primary fat-soluble antioxidant and comprises of a family of organic compounds including two isoforms, tocotrienol and tocopherol. Both tocopherol and tocotrienol appear in four isomers, α (alpha), β (beta), δ (delta), and γ (gamma). Unlike tocopherol, tocotrienol is an unsaturated form that features an isoprenoid side chain, making it easier to absorb and penetrate better into tissues with saturated fatty layers, including the liver and the brain (92). Tocotrienol was preferably issued to the liver, and α-tocotrienol is 40–60 times stronger than α-tocopherol in countering lipid peroxidation of liver microsomes (93). On the other hand, tocopherol is naturally lipophilic, protecting the polyunsaturated fatty acid compounds from peroxidation reactions, including lipoproteins, cellular membranes, and fat deposits (92). Interestingly, comparative studies on both vitamin E types showed that tocotrienol was far more potent than tocopherol in mitigating oxidative stress and inflammation (94, 95). Tocotrienol, as a free radical scavenger, can mitigate oxidative stress in metabolic disorders and protecting cellular functions (96). The δ-tocotrienol featured better bioavailability when supplemented at higher doses in healthy subjects, as reported by Qureshi et al. (97). Since most of the orally treated tocotrienol were metabolized inside the liver, these tests' outcomes suggested that tocotrienol supplements improved the obesity-stricken liver function and the energy spending of the entire body through improved oxidation of hepatic fatty acids (73).

### *In vitro* Evidence of Hepatoprotective Effect of Tocotrienol

Tocotrienol has been shown to have hypolipidemic properties in cells. The hypolipidemic property of tocotrienol is mediated through reduction of HMG-CoA (3-hydroxy-3-methyl-glutaryl-coenzyme A reductase), a key enzyme for the cholesterol biosynthesis (98, 99). *In vitro* research revealed that α-tocotrienol considerably decreased the total cholesterol (TC) compared to untreated SH-SY5Y cells and cells treated with α-tocopherol (100). A study using HepG2 cells treated with δ- and γ-tocotrienol indicated suppression of genes involved in lipid homeostasis including HMCR and APOB100 hence reducing TGs, TCs, and VLDL biosynthesis (101). Tocotrienol also has a powerful effect on decreasing adiposity as well as fat cell formation. It was postulated that γ-tocotrienol could decrease adipogenesis as the process shared the same pathway as in tumorigenesis. Fat cell development (adipogenesis) shares numerous similar signaling pathways with tumorigenesis; including the STAT, Wnt, mTOR signaling, and Akt autophagy pathways (98). Adipogenesis is defined as the differentiation of preadipocytes into mature adipocytes and increased accumulation and synthesis of intracellular TGs and lipid droplets (102). Zhao et al. employed hASCs as a human adipocyte model to identify the inhibitory effects of γ-tocotrienol on adipogenesis (74). The study found that γ-tocotrienol prevented adipogenesis in differentiating hASCs in an isomer- and dose-dependent manner. In several adipocyte cell lines, γ- and δ-tocotrienol were reported to exert anti-obesity characteristics through Bax-mediated mitochondrial or AMPK signaling pathways (103, 104). Based on the study of γ-tocotrienol effect on prostate cancer cells, activation of 5′ AMP-activated protein kinase (AMPK) signaling is vital for anti-adipogenic activity induced by γ-tocotrienol to elicit a cytotoxic effect on cancer cells (105). Nevertheless, inhibition of AMPK did not change the γ-tocotrienol-induced autophagy, implying that autophagy might not necessarily be directly related to the anti-adipogenic activity induced by γ-tocotrienol (73). These results showed that AMPK is a significant target for tocotrienol induced anti-adipogenesis in which the anti-adipogenic activity could be exhibited by its anti-proliferative capabilities. The AMPK is a modulator for lipid and cholesterol pathways thus, an excellent therapeutic target for treating NAFLD. On the other hand, Torabi et al. reported that *d*-δ-tocotrienol inhibited the differentiation of murine 3T3-F442A preadipocytes through the downregulation of peroxisome proliferator-activated receptor γ (PPARγ), a key regulator of adipocyte differentiation (99). Apart from anti-adipogenic property, tocotrienol also possesses anti-inflammatory property. In 2016, Kim et al. reported inhibition of nod-like receptor 3 (NLRP3) inflammasome in iJ774 macrophages by γ-tocotrienol thus attenuated adipose tissue inflammation and insulin resistance (106). Shen et al. reported δ-tocotrienol isolated from rice bran significantly inhibited inflammation via mitogen-activated protein kinase (MAPK) and PPAR signaling in lipopolysaccharide (LPS)-stimulated macrophages via inhibition of TNF-α, IFN-γ, IL-1β, and IL-6 (107, 108). The LPS stimulates the release of inflammatory cytokines, including IL-6, IL-1β, and TNF-α (109). The activation of nuclear factor kappa-light-chain-enhancer of activated B cells (NF-κB) and MAPK signaling were attenuated significantly in γ-tocotrienol-treated human adipocytes (107). Additionally, the δ-tocotrienol inhibited MAPK activation, which is essential for initiation of inflammation process, by inhibiting phosphorylation of p38, c-June N-terminal kinase (JNK), and extracellular signal-regulated kinase (110) in LPS-induced hASCs (107).Similar anti-inflammatory activity was exerted by δ-tocotrienol on TNF-α stimulated NF-κB activation in RAW 264.7 macrophages in a dose-dependent manner (111). In human gastric cells, γ-tocotrienol exhibited anti-cancer property via regulation of NF-κB signaling pathway. The aversion of NF-κB activation, an important pro-inflammatory marker involved in cell proliferation and apoptosis, subsequently halted tissue inflammation (112, 113).

### *In vivo* Evidence of Hepatoprotective Effect of Tocotrienol

Tocotrienol has a beneficial influence on reducing body fat or mass, plasma concentration of free fatty acid, cholesterol, and improving insulin tolerance in animal models and humans (73). Excessive amount of adipocytes secrete excess amount of adipokines and cytokines resulted in dysregulated lipid metabolism and then obesity therefore, fat cell apoptosis is necessary for inhibition of adipogenesis (73). Tocotrienol demonstrated an anti-adipogenic activity by causing adipocyte cell death (112). *In vivo* analysis of TRF supplementation with royal jelly in calorie restricted diet-fed rats indicated significant weight reduction and reduced expression of inflammatory cytokines (i.e. TNF-a and MCP1) as compared to TRF or royal jelly intervention alone (114). Kim et al. reported supplementation of γ-tocotrienol in Western-diet fed mice induced a profound decrease in TC and LDL levels apart from TG reduction by 50% (64). Furthermore, rice-bran tocotrienol inhibited adipogenesis in Western-diet fed mice via the upregulation of lipid metabolism genes, carnitine palmitoyltransferase 1A (*CPT1A*), and cytochrome P450 family 7 subfamily A member 1 (*CYP7A1*) (115). High-purity tocotrienols also possessed antiatherogenic property, where apolipoprotein E knockout mice fed with cholesterol-containing diet had reduced atherosclerotic lesion formation in the aortic root when compared to α-tocopherol treatment possibly through upregulation of *Slc27a1* gene (116). Since NAFLD and IR are highly associated with inflammation, many studies have shown tocotrienol as a potent anti-inflammatory inhibitor in animal models. In HF-fed mice supplemented with δ-tocotrienol at 400 mg/kg or 1600 mg/kg for 14 weeks, reduced expression of *tumor necrosis factor-alpha* (TNF-α) mRNA, a pro-inflammatory marker was observed (117). The authors suggested that the observed reduction in hepatic steatosis and serum TG levels when compared to HF-fed mice was due to reduced inflammation both in the liver and adipose tissue (117). Furthermore, treating T2DM in db/db mice with γ-tocotrienol delayed the progression of T2DM by suppressing the activation of NLRP3 inflammasome and associated inflammatory cytokines (106). Shen et al. observed better glucose homeostasis in HF-diet induce T2DM mice when treated with annatto-extracted tocotrienol by alleviating inflammatory response (118). Likewise, Kim et al. reported the supplementation of γ-tocotrienol in Western-diet fed mice reduced the expression level of pro-inflammatory mRNAs, including monocyte chemoattractant protein 1 (*Mcp1*), *Nlrp3, Tnf*α*, Il-1*β, and *cdc11* (119).

### Hepatoprotective Effect of Tocotrienol in Clinical Trials

**Figure 3:**
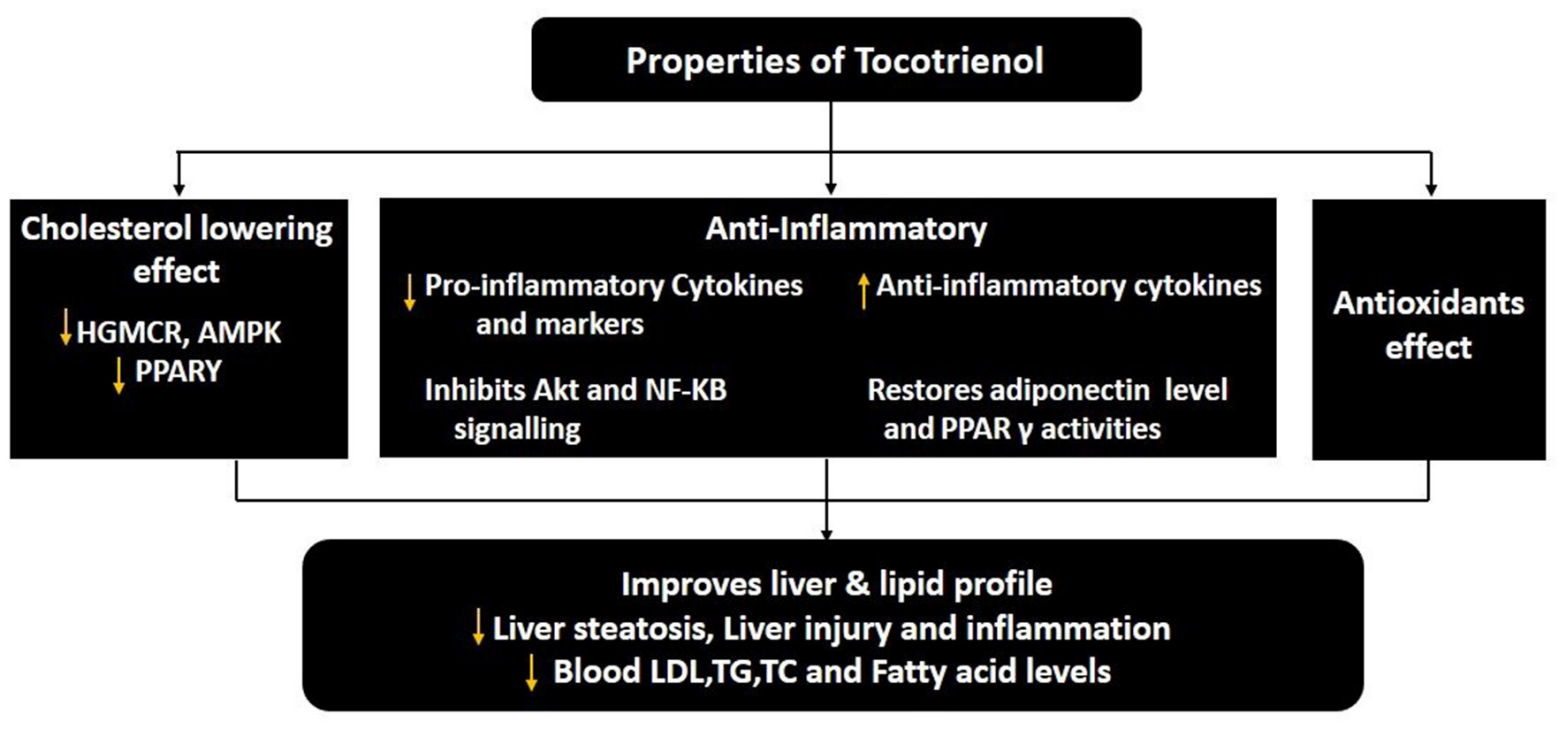
The pharmacological properties of tocotrienol for the treatment of pediatric NAFLD. ↑, increase or upregulate; ↓, decrease or downregulate; PPARγ, peroxisome proliferator-activated receptor γ; NF-κB, nuclear factor-κB; LDL, low-density lipoprotein; TG, triglyceride; TC, total cholesterol, ((125)).

Tocotrienol has anti-sclerotic and hypo-cholesterol impacts on humans (73). The tocotrienol-rich fraction (TRF) derived from palm oil was found to reduce total serum cholesterol, apolipoprotein B, LDL-cholesterol, and TG levels in patients with non-familial hypercholesterolemia (120). In a cross-sectional study (*n* = 200) of subjects with or without NAFLD, Ezaizi et al. reported a significant association between high-sensitivity C-reactive protein (hs-CRP) with NAFLD in Asian Indians (81). The hs-CRP is known to be associated with inflammation in the liver. Pervez et al. also reported decreased level of hs-CRP in addition to HOMA-IR, ALT and AST level in patients with NAFLD treated with δ-tocotrienol compared to placebo (121). Recently, Gao et al. reported the involvement of CYP4A11 in the development of NAFLD through ROS-induced lipid peroxidation and inflammation (122). Since CYP4A11 belongs to the CYP4 enzyme family involved in the metabolism of medium- and long-chain fatty acids, it is crucial in NAFLD pathogenesis. The patients with NAFLD were shown to have an increased amount of CYP4A11, possibly induced by a high-fat diet. The CYP4A11 increased ROS production during fatty acid oxidation, thus promoting oxidative stress. In a randomized controlled trial of 54 patients with diabetic nephropathy, the group receiving tocotrienol-rich vitamin E at 400 mg/day for 12 weeks had a significant reduction in serum creatinine levels and increased eGFR, hallmarks of inflammation, compared to placebo (123). To date, limited studies are available that documented the effect of tocotrienol on pediatric NAFLD. In a TONIC clinical trial for pediatric NAFLD, Lavine et al. (2011) found no significant difference in patients receiving either Vitamin E, metformin or placebo in terms of ALT level and histological features. Thus, designing clinical trials to test the effect of tocotrienol in the pediatric population should be properly examined due to the differences in several aspects that differentiate adult and pediatric NAFLD (124). Figure 3 summarizes the properties of tocotrienol as an adjunct therapy for the treatment of pediatric NAFLD.

## Conclusion

Despite high occurrence and rigorous research, NAFLD treatment remains an unfulfilled medical requirement. Presently, no specific pharmacological therapy was authorized for NAFLD. Nonetheless, patients are encouraged to improve their lifestyles through physical activities, diet, and managing related comorbidity (i.e., metabolic syndrome and obesity elements), which are supposed to reduce cardiovascular-related, hepatic morbidity in NASH patients. Nevertheless, patients often found that participating in physical activity and changing dietary habits are tough to achieve and maintain. Therefore, the characteristics of tocotrienol, which are easily found and exceptional acceptability to people, have made it a practical treatment choice for NAFLD patients. NAFLD clinical experiments showed a moderate improvement in histology and liver biochemistry induced by tocotrienol treatment. However, further monotherapy clinical trials and pharmacological assessments are still required to explain the fundamental molecular mechanisms of treatment. Also, possible unpleasant consequences of tocotrienol to identify the best daily intake needed for pediatric NAFLD. In conclusion, tocotrienol is recommended as a functional diet to be used in combination with existing diabetes and obesity regulation strategies for the treatment of pediatric NAFLD.

## Author Contributions

NM and ZA contributed to the conception of the idea for the manuscript. FA-B and AI performed literature search and drafted the manuscript. RR, NM, ZA, and KM critically revised the work. All authors contributed to the article and approved the submitted version.

## Conflict of Interest

The authors declare that the research was conducted in the absence of any commercial or financial relationships that could be construed as a potential conflict of interest.
